# Supplementary material for: Reaching Natural Growth: Light Quality Effects on Plant Performance in Indoor Growth Facilities
Source: Plants (Basel). 2020 Sep 27;9(10):1273. doi: 10.3390/plants9101273 (PMC7599614; doi:10.3390/plants9101273)
Supplement: Supplementary file 1 [file plants-09-01273-s001.pdf]

Table S1: Raw average values by measured trial for each treatment and specie. Plus minus values standard errors of these.

| **Species** | | | **Alnus** | | | | | **Ulmus** | | |
|---|---|---|---|---|---|---|---|---|---|---|
| **Trial\Treatment** | **Outdoor trial** | **6%B** | **25% B** | **35%B** | **62%B** | **Outdoor trial** | **6%B** | **25% B** | **35%B** | **62%B** |
| **Biomass and Morphology** | | | | | | | | | | |
| Height (cm) | 22.32±1.2 | 20.2±0.21 | 10.45±0.82 | 11.62±2.28 | 8.53±1.5 | 31.18±3.44 | 48.51±2.7 | 45.35±2.38 | 48.95±2.89 | 31.19±2.55 |
| Dry weight leaves (g) | 1.06±0.1 | 0.5±0.05 | 0.27±0.04 | 0.33±0.11 | 0.32±0.07 | 1.33±0.28 | 2.66±0.32 | 2.75±0.26 | 2.85±0.29 | 1.52±0.25 |
| Dry weight shoot (g) | 0.51±0.05 | 0.22±0.03 | 0.13±0.02 | 0.15±0.05 | 0.12±0.02 | 0.66±0.16 | 1.69±0.23 | 1.71±0.19 | 1.79±0.23 | 0.79±0.16 |
| Dry weight roots (g) | 0.52±0.05 | 0.58±0.07 | 0.17±0.03 | 0.17±0.05 | 0.3±0.06 | 0.55±0.13 | 1.98±0.24 | 2.5±0.28 | 2.4±0.33 | 1.45±0.36 |
| Total dry weight (g) | 2.09±0.19 | 1.3±0.13 | 0.57±0.09 | 0.64±0.2 | 0.74±0.15 | 2.54±0.56 | 6.34±0.77 | 6.96±0.66 | 7.05±0.82 | 3.76±0.75 |
| Root to Shoot ratio | 0.34±0.03 | 0.8±0.07 | 0.42±0.05 | 0.44±0.04 | 0.68±0.01 | 0.27±0.02 | 0.46±0.02 | 0.57±0.05 | 0.5±0.04 | 0.55±0.07 |
| SLA ($m^2 kg^{-1}$) | 34.76±1.24 | 36.41±2.99 | 33.61±0.99 | 35.49±2.45 | 31.67±1.18 | 28.35±0.85 | 28.45±0.79 | 23.08±0.62 | 24.23±0.92 | 25.05±0.78 |
| **Chlorophyll** | | | | | | | | | | |
| Chlorophyll a ($mg g^{-1}$) | 7.35±0.97 | 4.58±0.37 | 4.62±0.35 | 6.88±0.74 | 6.93±0.46 | 6.8±0.43 | 3.18±0.39 | 3.28±0.63 | 2.06±0.34 | 4.1±0.31 |
| Chlorophyll b ($mg g^{-1}$) | 1.45±0.25 | 1.02±0.11 | 1±0.07 | 1.49±0.15 | 1.45±0.11 | 1.54±0.09 | 0.71±0.09 | 0.74±0.15 | 0.45±0.07 | 0.88±0.09 |
| Chl a: b ratio | 1.94±0.18 | 1.68±0.04 | 1.99±0.18 | 2.9±0.21 | 2.56±0.11 | 1.15±0.09 | 0.98±0.09 | 1.06±0.17 | 0.72±0.11 | 1.11±0.08 |
| Carotenoids ($mg g^{-1}$) | 5.12±0.23 | 4.55±0.1 | 4.61±0.09 | 4.6±0.07 | 4.79±0.09 | 4.42±0.05 | 4.53±0.09 | 4.48±0.14 | 4.44±0.17 | 4.7±0.13 |
| Fv/Fm | 0.78±0.01 | 0.74±0.01 | 0.77±0.01 | 0.76±0.02 | 0.8±0 | 0.81±0 | 0.73±0.02 | 0.77±0.01 | 0.78±0.01 | 0.79±0.01 |
| **Standardized light** | | | | | | | | | | |
| Max photosynthesis ($CO_2$ μmol $m^{-2} s^{-1}$) | 14.35±1.48 | 10.22±1.52 | 6.78±1.05 | 11.6±1.33 | 12.31±1.21 | 12.87±1.9 | 9.01±0.68 | 11.82±0.36 | 11.51±0.5 | 12.68±0.95 |
| Initial slope | 0.044±0.001 | 0.037±0.002 | 0.026±0.004 | 0.038±0.004 | 0.041±0.005 | 0.045±0.002 | 0.043±0.003 | 0.042±0.002 | 0.04±0.001 | 0.05±0.006 |
| Dark respiration ($CO_2$ μmol $m^{-2} s^{-1}$) | −1.05±0.15 | −0.86±0.2 | −1.09±0.13 | −1.59±0.16 | −1.39±0.27 | −1.33±0.22 | −1.46±0.26 | −1.13±0.14 | −1.1±0.19 | −1.83±0.39 |
| Compensation point (PPFD μmol $m^{-2} s^{-1}$) | 22.67±4.33 | 21.67±3.76 | 41.33±4.29 | 38.67±2.33 | 29±2.65 | 24.33±2.85 | 25.83±3.76 | 25.5±2.64 | 25±3.46 | 28.17±1.38 |
| | | | | | | | | | | |
| **In−situ' light** | | | | | | | | | | |
| Max photosynthesis ($CO_2$ μmol $m^{-2} s^{-1}$) | 8.37±0.29 | 10.97±0.66 | 9.81±1.48 | 9.03±1.57 | 8.86±3.41 | 7.3±0.39 | 7.21±0.89 | 11.47±0.99 | 9.6±0.76 | 5.53±1.23 |
| Initial slope | 0.044±0.002 | 0.048±0.009 | 0.054±0.006 | 0.049±0.004 | 0.039±0.008 | 0.034±0.002 | 0.048±0.004 | 0.055±0.003 | 0.052±0.003 | 0.034±0.005 |
| Dark respiration ($CO_2$ μmol $m^{-2} s^{-1}$) | −1.74±0.07 | −1.54±0.37 | −1.58±0.24 | −1.45±0.21 | −1.13±0.32 | −1.27±0.15 | −1.16±0.13 | −1.3±0.12 | −1.33±0.15 | −1.11±0.23 |
| Compensation point (PPFD μmol $m^{-2} s^{-1}$) | 30.33±2.67 | 37.67±19.19 | 26.67±4.17 | 26.67±4.67 | 26.33±3.38 | 30.33±0.88 | 18.83±1.19 | 20.83±1.92 | 22.83±2.98 | 24.67±3.21 |
| **Species** | | | **Ocimum** | | | | | **Lactuca** | | |
| **Trial\Treatment** | **Outdoor** | **6%B** | **25% B** | **35%B** | **62%B** | **Outdoor** | **6%B** | **25% B** | **35%B** | **62%B** |

| | **trial** | | | | | **trial** | | | | |
|---|---|---|---|---|---|---|---|---|---|---|
| **Biomass and Morphology** | | | | | | | | | | |
| Height (cm) | 21.17±1.38 | 30.42±0.89 | 26.09±0.79 | 28.15±1.48 | 23.99±1.03 | – | – | – | – | – |
| Dry weight leaves (g) | 1.18±0.15 | 2.32±0.15 | 2.03±0.13 | 2.12±0.18 | 1.72±0.14 | 8.97±0.49 | 8.98±0.55 | 10.01±0.53 | 10.17±0.47 | 10.42±0.49 |
| Dry weight shoot (g) | 0.37±0.04 | 0.78±0.06 | 0.62±0.04 | 0.75±0.07 | 0.51±0.05 | - | - | - | - | - |
| Dry weight roots (g) | 0.36±0.06 | 1.93±0.28 | 1.02±0.14 | 0.98±0.11 | 1.24±0.24 | 3.21±0.3 | 10.65±1.11 | 9.33±1.46 | 9.37±1.36 | 11.5±1.18 |
| Total dry weight (g) | 1.91±0.23 | 5.03±0.31 | 3.67±0.27 | 3.85±0.33 | 3.47±0.3 | 12.18±0.77 | 19.64±1.53 | 19.34±1.83 | 19.54±1.72 | 21.91±1.43 |
| Root to Shoot ratio | 0.24±0.02 | 0.7±0.12 | 0.37±0.04 | 0.34±0.03 | 0.63±0.14 | 0.35±0.02 | 1.18±0.08 | 0.9±0.12 | 0.88±0.11 | 1.1±0.11 |
| SLA ($m^2$ $kg^{-1}$) | 20.7±1.32 | 13.86±0.77 | 18.09±0.84 | 17.09±0.85 | 19.56±0.74 | 25.07±1.5 | 39.57±2.23 | 33.26±1.94 | 24.45±1.38 | 32.23±1.03 |
| **Chlorophyll** | | | | | | | | | | |
| Chlorophyll a (mg $g^{-1}$) | 2.19±0.29 | 2.33±0.14 | 3.51±0.2 | 2.99±0.26 | 4.29±0.37 | 2.51±0.53 | 2.06±0.24 | 2.84±0.42 | 1.8±0.13 | 3.99±0.51 |
| Chlorophyll b (mg $g^{-1}$) | 0.54±0.08 | 0.49±0.03 | 0.74±0.05 | 0.7±0.08 | 0.93±0.09 | 0.57±0.12 | 0.35±0.04 | 0.5±0.08 | 0.33±0.02 | 0.71±0.09 |
| Chl a: b ratio | 0.55±0.08 | 0.66±0.05 | 0.96±0.04 | 0.77±0.04 | 1.09±0.1 | 0.68±0.14 | 0.99±0.05 | 1.05±0.13 | 0.73±0.05 | 1.43±0.15 |
| Carotenoids (mg $g^{-1}$) | 4.08±0.05 | 4.8±0.19 | 4.81±0.08 | 4.39±0.15 | 4.62±0.06 | 4.38±0.33 | 5.93±0.16 | 5.84±0.19 | 5.46±0.09 | 5.62±0.2 |
| Fv/Fm | 0.77±0.02 | 0.83±0.01 | 0.82±0 | 0.84±0 | 0.84±0 | 0.85±0.01 | 0.8±0.01 | 0.84±0.01 | 0.84±0.01 | 0.84±0.01 |
| **Standardized light** | | | | | | | | | | |
| Max photosynthesis ($CO_2$ µmol $m^{-2}$ $s^{-1}$) | 10.18±2.77 | 19.17±0.95 | 20.43±1.1 | 22.52±0.49 | 23.13±1 | 4.63±0.99 | 14.62±1.6 | 11.99±1.87 | 17.28±2.34 | 17.67±3.01 |
| Initial slope | 0.043±0.002 | 0.047±0.003 | 0.044±0.003 | 0.045±0.001 | 0.053±0.001 | 0.043±0.003 | 0.045±0.004 | 0.042±0.004 | 0.044±0.003 | 0.052±0.004 |
| Dark respiration ($CO_2$ µmol $m^{-2}$ $s^{-1}$) | −3.99±0.29 | −2.44±0.23 | −2.02±0.13 | −1.99±0.12 | −2.11±0.06 | −1.12±0.15 | −1.99±0.22 | −1.68±0.18 | −1.96±0.22 | −2.29±0.27 |
| Compensation point (PPFD µmol $m^{-2}$ $s^{-1}$) | 91.33±3.84 | 49±5.22 | 45.5±4.43 | 42.5±1.65 | 36.17±2.14 | 16±2.08 | 41±4.15 | 40.33±7.82 | 41.5±4.93 | 38.67±3.03 |
| **In–situ' light** | | | | | | | | | | |
| Max photosynthesis ($CO_2$ µmol $m^{-2}$ $s^{-1}$) | 6.73±0.3 | 13.78±0.92 | 17.65±1.42 | 17.24±0.98 | 16±1.07 | 5.77±0.65 | 7.11±1.67 | 10.48±1.59 | 12.08±1.5 | 8.49±2.04 |
| Initial slope | 0.037±0.001 | 0.059±0.003 | 0.059±0.004 | 0.061±0.003 | 0.057±0.003 | 0.038±0.002 | 0.045±0.007 | 0.053±0.003 | 0.055±0.002 | 0.047±0.005 |
| Dark respiration ($CO_2$ µmol $m^{-2}$ $s^{-1}$) | −2.8±0.21 | −1.78±0.14 | −1.98±0.23 | −1.95±0.26 | −2.1±0.11 | −1.85±0.17 | −1.33±0.25 | −1.56±0.19 | −1.81±0.12 | −1.56±0.22 |
| Compensation point (PPFD µmol $m^{-2}$ $s^{-1}$) | 76.33±3.38 | 28.17±2.61 | 33±4.38 | 31.67±5.63 | 34.67±3.33 | 38.33±6.12 | 23.67±3.29 | 27.83±4.61 | 30.83±2.43 | 24.5±3.27 |

Table S1 (continuation): Raw average values by measured trial for each treatment and specie. Plus minus values standard errors of these

| **Species** | **Melissa** | **Raphanus** |
|---|---|---|

| Trial\Treatment | Outdoor trial | 6%B | 25% B | 35%B | 62%B | Outdoor trial | 6%B | 25% B | 35%B | 62%B |
|---|---|---|---|---|---|---|---|---|---|---|
| **Biomass and Morphology** | | | | | | | | | | |
| Height (cm) | 26.75±0.76 | 22.05±1.38 | 17.51±1.58 | 23.48±0.5 | 10.97±0.59 | 5.84±0.19 | 8.62±0.56 | 6.41±0.24 | 6.73±0.61 | 5.43±0.41 |
| Dry weight leaves (g) | 2.93±0.48 | 2.85±0.39 | 2.91±0.43 | 3.27±0.25 | 1.58±0.13 | 2.35±0.18 | 1.88±0.15 | 1.86±0.15 | 1.61±0.13 | 1.46±0.09 |
| Dry weight shoot (g) | – | – | – | – | – | 0.53±0.06 | 0.6±0.07 | 0.51±0.04 | 0.57±0.08 | 0.35±0.03 |
| Dry weight roots (g) | 1.73±0.33 | 1.92±0.28 | 1.82±0.23 | 1.94±0.19 | 1.11±0.13 | 4.41±0.51 | 4.97±0.24 | 6.14±0.74 | 5.13±0.44 | 3.3±0.15 |
| Total dry weight (g) | 4.67±0.81 | 4.77±0.65 | 4.73±0.63 | 5.21±0.43 | 2.69±0.25 | 7.3±0.67 | 7.46±0.41 | 8.52±0.87 | 7.31±0.56 | 5.12±0.18 |
| Root to Shoot ratio | 0.58±0.03 | 0.68±0.05 | 0.72±0.07 | 0.59±0.03 | 0.7±0.05 | 1.52±0.13 | 2.16±0.13 | 2.56±0.22 | 2.44±0.17 | 1.92±0.13 |
| SLA ($m^2$ $kg^{-1}$) | 38.1±2.97 | 31.28±1.6 | 29.08±0.91 | 31.64±1.49 | 35.73±1.02 | 23.71±0.98 | 27.15±0.99 | 31.13±2.12 | 30.57±0.97 | 27.43±0.77 |
| **Chlorophyll** | | | | | | | | | | |
| Chlorophyll a (mg $g^{-1}$) | 8.04±1.54 | 5.57±0.65 | 4.58±0.7 | 5.27±0.39 | 6.76±0.29 | 2.72±0.8 | 3.24±0.29 | 5.08±0.71 | 3.48±0.39 | 4.84±0.51 |
| Chlorophyll b (mg $g^{-1}$) | 1.82±0.3 | 1.31±0.15 | 1.03±0.15 | 1.22±0.09 | 1.54±0.1 | 0.67±0.16 | 0.74±0.06 | 1.17±0.15 | 0.79±0.08 | 1.16±0.07 |
| Chl a: b ratio | 1.94±0.39 | 1.79±0.15 | 1.5±0.21 | 1.71±0.1 | 2.07±0.04 | 0.63±0.18 | 1.09±0.06 | 1.49±0.19 | 1.2±0.12 | 1.55±0.08 |
| Carotenoids (mg $g^{-1}$) | 4.35±0.18 | 4.25±0.05 | 4.43±0.04 | 4.31±0.05 | 4.43±0.1 | 3.92±0.24 | 4.35±0.1 | 4.32±0.14 | 4.43±0.16 | 4.13±0.25 |
| Fv/Fm | 0.81±0.01 | 0.78±0.02 | 0.82±0 | 0.78±0.01 | 0.82±0 | 0.83±0.01 | 0.82±0.01 | 0.83±0.01 | 0.82±0.01 | 0.83±0.01 |
| **Standardized light** | | | | | | | | | | |
| Max photosynthesis (PPFD μmol $m^{-2}$ $s^{-1}$) | 19.11±2.88 | 13.64±1.24 | 17.2±0.81 | 15.78±1.59 | 15.96±1.07 | 10.31±1.25 | 24.72±1.12 | 18.19±1.52 | 22.66±1.72 | 24.02±2.44 |
| Initial slope | 0.054±0.004 | 0.041±0.003 | 0.048±0.002 | 0.04±0.003 | 0.051±0.006 | 0.053±0.002 | 0.053±0.002 | 0.057±0.004 | 0.052±0.002 | 0.062±0.005 |
| Dark respiration ($CO_2$ μmol $m^{-2}$ $s^{-1}$) | −1.08±0.08 | −1.21±0.2 | −1.4±0.17 | −1.42±0.11 | −2.11±0.56 | −1.23±0.11 | −2.19±0.15 | −1.63±0.24 | −1.85±0.14 | −2.36±0.23 |
| Compensation point (PPFD μmol $m^{-2}$ $s^{-1}$) | 18.33±0.88 | 26±3.81 | 27.67±3.7 | 33.67±2.03 | 35±4.23 | 20.33±2.85 | 40.67±3.22 | 26.67±5.3 | 34.83±2.68 | 35.17±3.05 |
| **In–situ' light** | | | | | | | | | | |
| Max photosynthesis (PPFD μmol $m^{-2}$ $s^{-1}$) | 11.08±0.33 | 12.34±1.87 | 14.29±0.9 | 16.26±0.86 | 11.88±1.54 | 13.77±1.97 | 12.57±2.16 | 14.81±2.41 | 14.59±1.14 | 11.46±1.51 |
| Initial slope | 0.051±0.004 | 0.064±0.003 | 0.067±0.002 | 0.06±0.004 | 0.056±0.003 | 0.046±0.003 | 0.069±0.004 | 0.068±0.003 | 0.069±0.002 | 0.055±0.005 |
| Dark respiration ($CO_2$ μmol $m^{-2}$ $s^{-1}$) | −1.63±0.09 | −1.47±0.14 | −1.51±0.14 | −1.6±0.11 | −1.79±0.16 | −1.05±0.01 | −1.64±0.1 | −1.53±0.32 | −1.56±0.16 | −1.57±0.14 |
| Compensation point (PPFD μmol $m^{-2}$ $s^{-1}$) | 24.33±1.2 | 20.5±2.19 | 21±2.8 | 26±3.49 | 26.5±3.27 | 22.33±1.33 | 20.67±1.38 | 21±4.77 | 21.5±2.38 | 25.17±6.28 |

Table S1 (continuation): Raw average values by measured trial for each treatment and specie. Plus minus values standard errors of these

| Species | | | Triticum | | |
|---|---|---|---|---|---|
| Trial\Treatment | Outdoor | 6%B | 25% B | 35%B | 62%B |

| | **trial** | | | | |
|---|---|---|---|---|---|
| **Biomass and Morphology** | | | | | |
| Height(cm) | 52.41±1.37 | 51.9±0.98 | 45.73±2.73 | 46.78±0.63 | 39.27±1.19 |
| Dry weight leaves (g) | 12.26±0.46 | 10.45±0.36 | 9.92±0.44 | 8.74±0.56 | 6.8±0.37 |
| Dry weight shoot (g) | – | – | – | – | – |
| Dry weight roots (g) | 21.04±2.43 | 81.74±7.21 | 82.1±8.96 | 56.6±5.24 | 93.12±6.46 |
| Total dry weight (g) | 33.3±2.34 | 92.19±7.36 | 92.02±8.77 | 65.34±5.38 | 99.91±6.53 |
| Root to Shoot ratio | 1.76±0.25 | 7.78±0.62 | 8.82±1.2 | 6.79±0.7 | 14.21±1.13 |
| SLA ($m^2$ $kg^{-1}$) | 31.45±0.67 | 20.07±0.63 | 24.92±1.94 | 26.93±1.49 | 24.14±0.81 |
| **Chlorophyll** | | | | | |
| Chlorophyll a (mg $g^{-1}$) | 7.12±0.44 | 2.88±0.34 | 4.97±0.43 | 4.11±0.54 | 5.45±0.52 |
| Chlorophyll b (mg $g^{-1}$) | 1.89±0.07 | 0.67±0.09 | 1.15±0.11 | 0.98±0.17 | 1.14±0.14 |
| Chl a: b ratio | 1.45±0.05 | 0.94±0.08 | 1.65±0.07 | 1.18±0.13 | 1.58±0.11 |
| Carotenoids (mg $g^{-1}$) | 3.75±0.12 | 4.37±0.14 | 4.34±0.1 | 4.38±0.19 | 4.89±0.16 |
| Fv/Fm | 0.83±0 | 0.79±0.01 | 0.81±0.01 | 0.82±0.02 | 0.83±0 |
| **Standardized light** | | | | | |
| Max photosynthesis ($CO_2$ μmol $m^{-2}$ $s^{-1}$) | 16.92±1.13 | 13.39±0.78 | 14.52±2.48 | 13.04±1.4 | 15.44±1.37 |
| Initial slope | 0.056±0.004 | 0.058±0.009 | 0.054±0.006 | 0.046±0.006 | 0.071±0.005 |
| Dark respiration ($CO_2$ μmol $m^{-2}$ $s^{-1}$) | −1.5±0.11 | −1.95±0.4 | −1.72±0.31 | −1.42±0.16 | −2.1±0.25 |
| Compensation point (PPFD μmol $m^{-2}$ $s^{-1}$) | 23±2 | 28.83±6.52 | 26.17±3.49 | 30±4.97 | 22.83±2.97 |
| **In−situ' light** | | | | | |
| Max photosynthesis ($CO_2$ μmol $m^{-2}$ $s^{-1}$) | 9.12±0.47 | 9.82±1.93 | 12.62±0.87 | 10.27±1.82 | 9.73±2.83 |
| Initial slope | 0.044±0 | 0.064±0.01 | 0.073±0.003 | 0.059±0.009 | 0.07±0.021 |
| Dark respiration ($CO_2$ μmol $m^{-2}$ $s^{-1}$) | −1.9±0.05 | −1.42±0.26 | −1.61±0.3 | −1.31±0.26 | −1.9±0.41 |
| Compensation point (PPFD μmol $m^{-2}$ $s^{-1}$) | 35±0 | 21.67±2.53 | 19.17±3.57 | 19±2.24 | 21.17±3.39 |

Table S1 (continuation): Raw average values by measured trial for each treatment and specie. Plus minus values standard errors of these

| **Factors** | **Fix factors** | **Random factors** |
|---|---|---|
| | **Light quality** | **Specie** |
| **Variable** | | |
| **Biomass and Morphology** | | |
| Height (cm) | $< 2.2\times10^{-16}$ | $< 2.2x10^{-16}$ |
| Dry weight leaves (g) | $3.54x10^{-05}$ | $< 2.2x10^{-16}$ |
| Dry weight shoot (g) | $1.67x10^{-09}$ | $< 2.2x10^{-16}$ |
| Dry weight roots (g) | $7.91x10^{-08}$ | $< 2.2x10^{-16}$ |
| Total dry weight | $4.95x10^{-09}$ | $< 2.2x10^{-16}$ |
| Root to Shoot ratio | $5.88x10^{-07}$ | $< 2.2x10^{-16}$ |
| SLA ($m^2$ $kg^{-1}$) | 0.4753 | $< 2.2x10^{-16}$ |
| | | |
| **Chlorophyll** | | |
| Chlorophyll a (mg $g^{-1}$) | $5.73x10^{-11}$ | $< 2.2x10^{-16}$ |
| Chlorophyll b (mg $g^{-1}$) | $8.51x10^{-11}$ | $< 2.2x10^{-16}$ |
| Chl a: b ratio | $2.37x10^{-05}$ | $< 2.2x10^{-16}$ |
| Carotenoids (mg $g^{-1}$) | $7.59x10^{-13}$ | $< 2.2x10^{-16}$ |
| Fv/Fm | $4.02x10^{-08}$ | $< 2.2x10^{-16}$ |
| | | |
| **Standardized light** | | |
| Max photosynthesis ($CO_2$ µmol $m^{-2}$ $s^{-1}$) | $3.73x10^{-05}$ | $< 2.2x10^{-16}$ |
| Initial slope | $2.54x10^{-07}$ | $< 2.2x10^{-16}$ |
| Dark respiration ($CO_2$ µmol $m^{-2}$ $s^{-1}$) | $< 2.2x10^{-16}$ | $< 2.2x10^{-16}$ |
| Compensation point (PPFD µmol $m^{-2}$ $s^{-1}$) | 0.003218 | $< 2.2x10^{-16}$ |
| | | |
| **In–situ' light** | | |
| Max photosynthesis ($CO_2$ µmol $m^{-2}$ $s^{-1}$) | $5.66x10^{-07}$ | $< 2.2x10^{-16}$ |
| Initial slope | $8.14x10^{-07}$ | $1.00x10^{-04}$ |
| Dark respiration ($CO_2$ µmol $m^{-2}$ $s^{-1}$) | – | $< 2.2x10^{-16}$ |
| Compensation point (PPFD µmol $m^{-2}$ $s^{-1}$) | 0.003684 | $< 2.2x10^{-16}$ |

Table S2: P–values for the different measured traits in both experiments using normalized data.

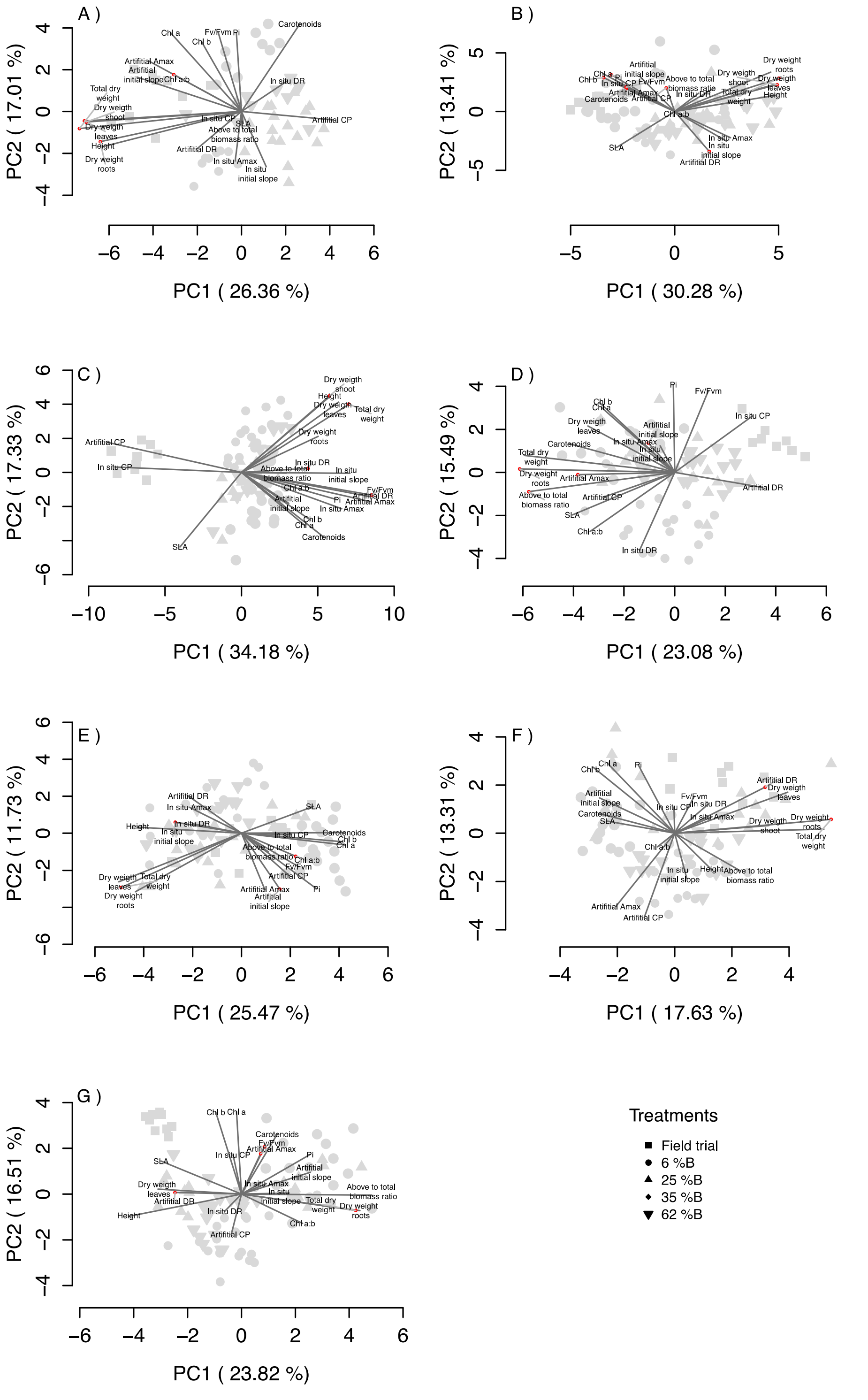


**Figure S1.** Principal component analysis (PCA) of the measured traits of each specie: A) *Alnus*, B) *Ulmus*, C) *Ocimum*, D) *Lactuca*, E) *Melissa*, F) *Raphanus* and G) *Triticum*, grown under 6% B, 25% B, 35% B and 62% B light together with the importance of the different measured traits in each specie. Lighter point (n=18) corresponds to a plant in each different treatment (See legend), and solid arrows to each measured traits.

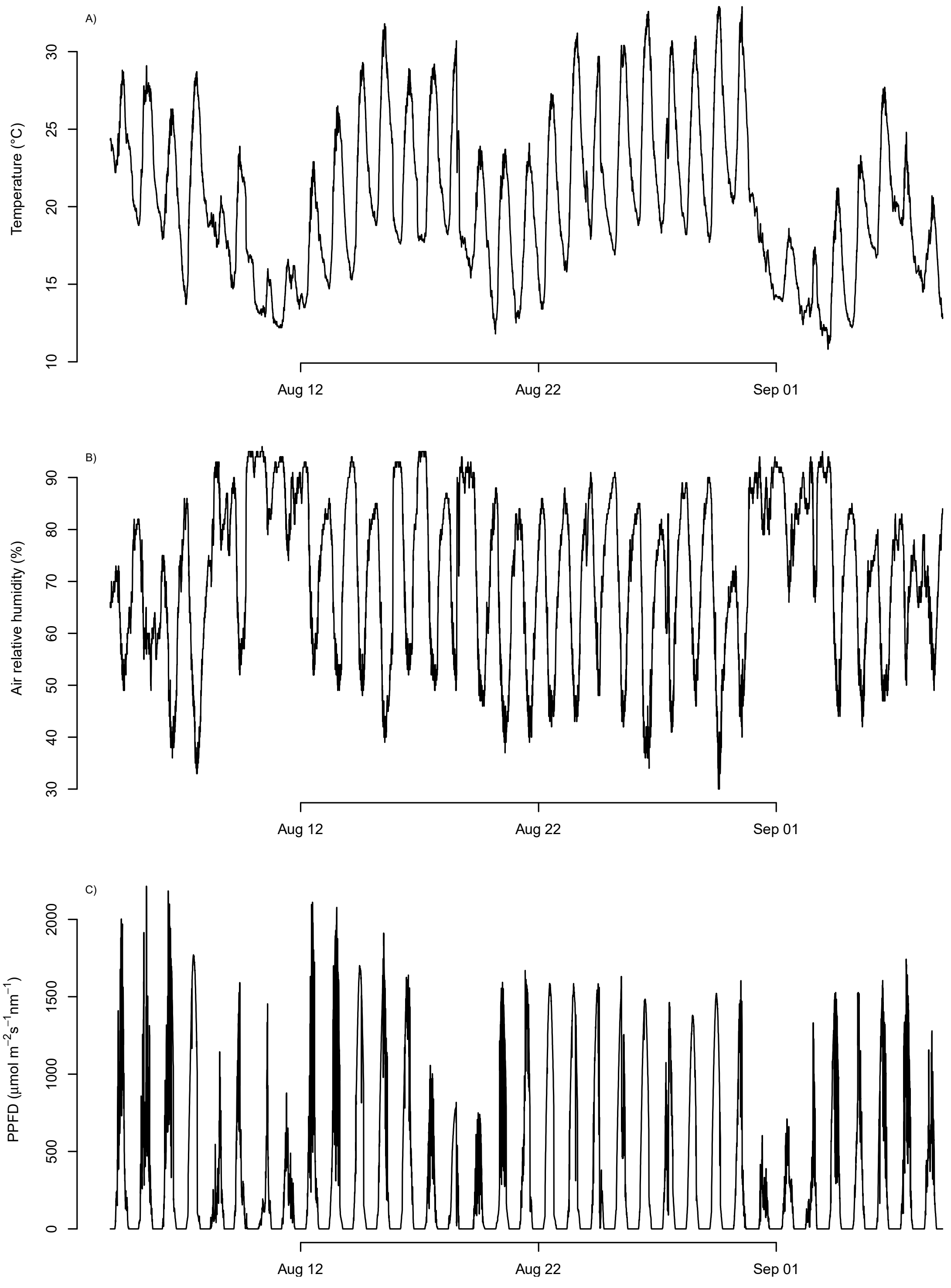


**Figure S2.** Environmental conditions of temperature (**A**), air relative humidity (**B**) and light intensity as PPFD (**C**) from the field trial at the botanical garden of the University of Basel, Switzerland.
